# A functional genomics screen identifies an Importin-α homolog as a regulator of stem cell function and tissue patterning during planarian regeneration

**DOI:** 10.1186/s12864-015-1979-1

**Published:** 2015-10-12

**Authors:** Amy Hubert, Jordana M. Henderson, Martis W. Cowles, Kelly G. Ross, Matthew Hagen, Christa Anderson, Claudia J. Szeterlak, Ricardo M. Zayas

**Affiliations:** Department of Biology, San Diego State University, San Diego, CA 92182-4614 USA; Department of Biological Sciences, Southern Illinois University Edwardsville, Edwardsville, IL 62026-0001 USA; Biological and Medical Informatics Research Center, San Diego State University, San Diego, CA 92182-4614 USA

**Keywords:** Neoblasts, Patterning, Importin, Functional genomics, *Schmidtea mediterranea*

## Abstract

**Background:**

Planarians are renowned for their regenerative capacity and are an attractive model for the study of adult stem cells and tissue regeneration. In an effort to better understand the molecular mechanisms underlying planarian regeneration, we performed a functional genomics screen aimed at identifying genes involved in this process in *Schmidtea mediterranea*.

**Methods:**

We used microarrays to detect changes in gene expression in regenerating and non-regenerating tissues in planarians regenerating one side of the head and followed this with high-throughput screening by in situ hybridization and RNAi to characterize the expression patterns and function of the differentially expressed genes.

**Results:**

Along with five previously characterized genes (*Smed-cycD*, *Smed-morf41/mrg-1*, *Smed-pdss2/dlp1, Smed-slbp*, and *Smed-tph*), we identified 20 additional genes necessary for stem cell maintenance (*Smed-sart3*, *Smed-smarcc-1*, *Smed-espl1*, *Smed-rrm2b-1*, *Smed-rrm2b-2*, *Smed-dkc1*, *Smed-emg1*, *Smed-lig1*, *Smed-prim2*, *Smed-mcm7*, and a novel sequence) or general regenerative capability (*Smed-rbap46/48-2*, *Smed-mcm2*, *Smed-ptbp1*, and *Smed-fen-1*) or that caused tissue-specific defects upon knockdown (*Smed-ddc*, *Smed-gas8*, *Smed-pgbd4*, and *Smed-b9d2*). We also found that a homolog of the nuclear transport factor Importin-α plays a role in stem cell function and tissue patterning, suggesting that controlled nuclear import of proteins is important for regeneration.

**Conclusions:**

Through this work, we described the roles of several previously uncharacterized genes in planarian regeneration and implicated nuclear import in this process. We have additionally created an online database to house our in situ and RNAi data to make it accessible to the planarian research community.

**Electronic supplementary material:**

The online version of this article (doi:10.1186/s12864-015-1979-1) contains supplementary material, which is available to authorized users.

## Background

The freshwater planarian *Schmidtea mediterranea* has the remarkable ability to regenerate any part of its body following amputation, and uninjured animals constantly replace cells in the differentiated tissues through a homeostatic process (reviewed in [1, 2]). Planarians owe their remarkable regenerative capacity to a pool of adult stem cells called neoblasts [3]. Recent studies have detected heterogeneity within the neoblast population [4, 5], demonstrating that some of these cells are pluripotent and capable of producing differentiated cells of any type [6], while others are specialized progenitors of particular cell types [4, 7–11]. Following injury, the neoblasts divide and migrate toward the wound site [12], where the regeneration blastema forms in the event of tissue loss. Neoblast progeny then differentiate into the appropriate cell types and integrate with the pre-existing tissue, and regeneration culminates with full restoration of function. Many of the genes underlying these processes are evolutionarily conserved [13–15]; thus, planarians are an excellent model for the study of tissue regeneration and adult stem cell maintenance and function, and studying planarian homologs of genes involved in human diseases can provide insight into those genes’ functions that could be applied toward finding new treatments.

Benefits to using planarians as a model system are the ability to do high throughput screens and the availability of protocols to analyze gene expression and function. Available tools include microarrays and mRNA sequencing to study changes in gene expression, optimized in situ hybridization protocols to identify patterns of expression in specific tissues, and RNA interference (RNAi) for assessing function. Using these techniques, planarian researchers have begun characterizing genes necessary for stem cell function and tissue regeneration. For example, high-throughput sequencing of transcriptomes of sorted stem cells and post-mitotic progeny has identified many genes expressed in neoblasts that may contribute to maintaining their pluripotency [13–17]. Other screens identified genes required for the initial response to wounding [18] or for regeneration of specific tissue types [10, 19–21]. Finally, smaller scale studies have shown that patterning of the regenerating tissues is guided in large part by conserved developmental pathways, including Wnt/β-catenin, BMP, and Slit-Robo signaling [22–24]. Questions still remain, however, regarding the full complement of genes required for planarian regeneration and how various cell types pattern relative to one another and the non-regenerating tissue.

We performed a functional genomics screen aimed at identifying previously uncharacterized genes involved in planarian regeneration. We amputated one side of the head and used microarrays to analyze changes in gene expression in the regeneration blastema as well as in the non-regenerating side of the head. In contrast to other screens that have examined differential gene expression during regeneration of the entire head [25, 26], our strategy allowed us to additionally look for genes required for repair (as opposed to de novo production) of tissues, including the central nervous system (CNS) and for signaling between the new and old tissue. We next performed large scale in situ hybridization experiments to examine the expression patterns of the differentially expressed genes, and based on these patterns and homology, we chose 156 genes to knock down by RNAi. We identified 25 genes required for various aspects of planarian regeneration, five of which have also been functionally characterized by other labs [21, 27–30]. The 20 newly characterized genes included those encoding homologs of the RNA binding protein SART3 and chromatin remodeler SMARCC-1, which were necessary for neoblast maintenance, *Smed-ddc*, which was needed for production of photoreceptor pigment, and *Smed-pgdb4*, which resulted in loss of the pharynx upon knockdown. We also identified a homolog of nuclear transport factor Importin-α and found it to be necessary for stem cell function, differentiation, and patterning of regenerating tissues relative to the midline. This is notable because it suggests that regulated nuclear import of proteins may play a role in regeneration. The results of this screen further our understanding of the molecular control of planarian regeneration by pinpointing additional genes involved in the animal’s remarkable ability to replace its tissues. We have created a publicly accessible database to house our in situ and RNAi results, making these data available to the planarian research community.

## Methods

### Planarian care

Asexual *Schmidtea mediterranea* from the CIW4 strain were maintained in 1X Montjuïc salts and fed homogenized calf liver. Animals were starved for 1 week prior to use in experiments. We used worms 1–2 mm in length for in situ hybridization and 3–5 mm for RNAi experiments.

### Clones and accession numbers

Constructs available in a collection of cDNA clones [31] were pulled from glycerol stocks, and inserts were subcloned into pJC53.2 [32] for use in RNAi experiments. Other sequences were directionally cloned into pJC53.2 using gene-specific primers to amplify from cDNA. Accession numbers or primers for each clone used in the screen are listed in Additional file 3 and Additional file 4. Clones used for in situ markers or follow-up experiments include *Smed-pc2* [GenBank:BK007043], *Smed-cintillo* [GenBank:AY067542], *Smed-inx* [GenBank:DN303464], *Smed-slit* [GenBank:DQ336176], *Smed-laminin* [GenBank:DN293829] and *Smed-ima-2* (3′ read) [GenBank:HO007189] and (5′ read) [GenBank:DN308983].

### Microarrays

**Fig. 1:**
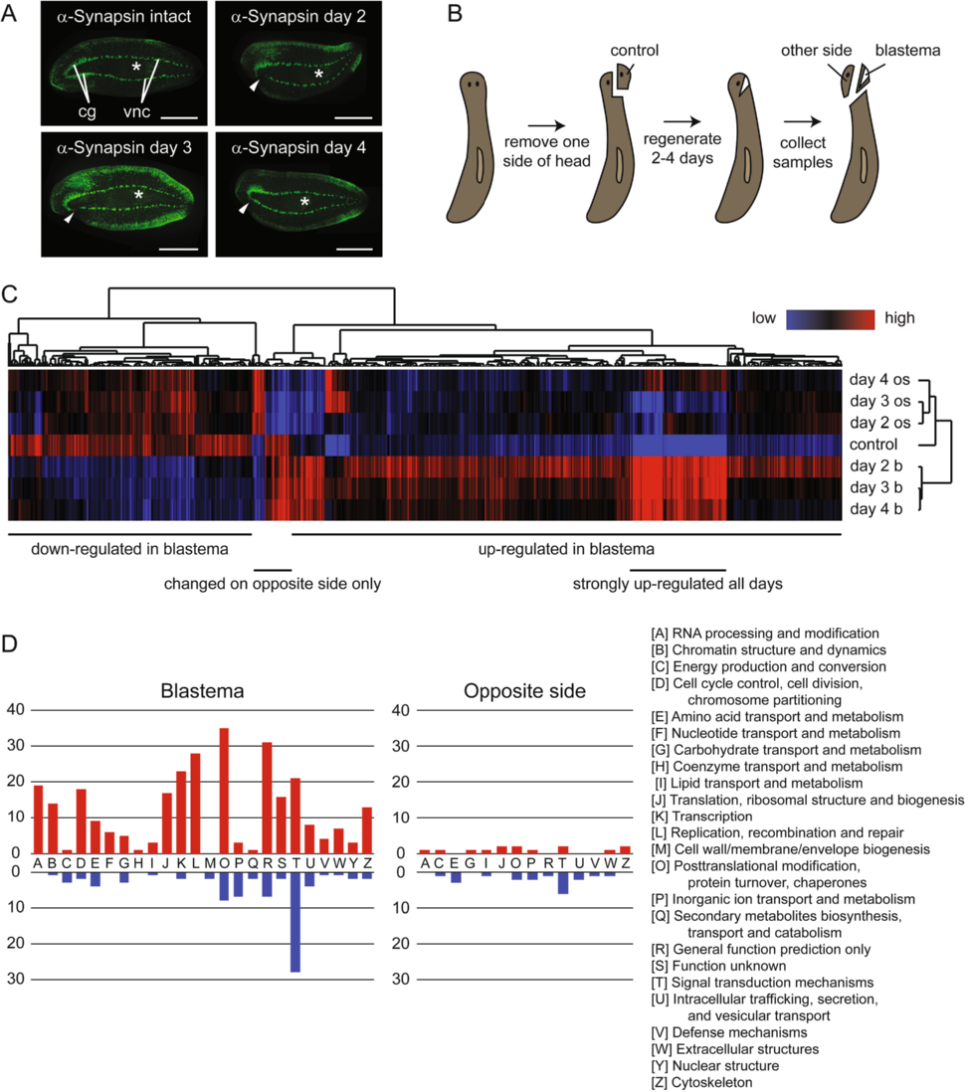
Identification of genes differentially expressed during head repair and regeneration. **a** Half-head regeneration. Anti-Synapsin antibody was used to label the central nervous system of intact animals and animals that had regenerated for two to 4 days following half-head amputation. Samples were imaged from the ventral side with anterior to the left, and the amputated portion of the head in regenerates is toward the bottom. cg = cephalic ganglia; vnc = ventral nerve cords. Asterisks mark the pharynx, and triangles point to where the nervous system is regenerating in the blastema. Scale bar = 0.5 mm. **b** Sample collection strategy. One side of the head was removed from intact animals and saved as the non-regenerating control sample. After two to four days of regeneration, samples were collected from the blastema and non-regenerating opposite side of the head. **c** Heat map summary of changes in gene expression in the blastema and other side of the head during a time course of half-head regeneration. Low expression is represented in blue and high in red. Control = non-regenerating samples. Day 2 b, day 3 b, and day 4 b = blastema samples collected on day two, three or four of regeneration. Day 2 os, day 3 os, and day 4 os = samples collected from the opposite side of the head on day two, three, or four of regeneration. **d** Categorization of differentially expressed genes based on the function of their homologs in other species using Clusters of Orthologous Groups. Red bars show the number of upregulated genes in each functional group in either the blastema or opposite side tissue relative to control. Blue bars represent downregulated genes

Samples were collected from intact and regenerating animals as described in the Results section (see Fig. 1). Four separate biological replicates were processed for control and each of the blastema samples across the time course, and two were processed for the “opposite side” samples. Total RNA was extracted with Trizol reagent (Invitrogen), treated with Turbo DNA-free DNase (Ambion), then further purified using the Qiagen RNeasy MinElute Cleanup kit. cDNA was synthesized with the SuperScript Double-Stranded cDNA Synthesis kit (Invitrogen). cDNA labeling and array hybridization were carried out by the GeneChip core at the University of California San Diego. Each sample was hybridized to a separate Nimblegen array (design 2007-11-06_Smed_ESTs_4_exp [33, 34]) containing probes for 16,797 ESTs representing approximately 11,584 unique loci, with probes spotted in duplicate to provide technical replication.

### Microarray data analysis

Nimblescan software was used to perform Robust Multi-array Analysis (RMA) for quantile normalization and probe-level summarization of the expression data, with normalization occurring first between arrays of the same sample type and then across all samples. Background correction was applied during normalization. Probe sets with values below 32 (5 on a log_2_ scale) in all samples were omitted from further analysis, and expression of ESTs representing the same genomic locus was averaged to give one value per locus for each sample. The Limma Bioconductor program [35] was used to fit the expression data to a linear model and make pairwise contrasts between the control and each of the other six sample types (blastema and opposite side tissue from two-, three-, and four-day regenerates). The Benjamini and Hochberg correction [36] was applied to adjust *p*-values to control the false discovery rate for multiple comparisons (F-test *p* ≤ 0.05). The microarray data have been deposited in the Gene Expression Omnibus (accession GSE62551).

### Heat map generation

Gene Cluster 3.0 [37] was used to produce the heat map of microarray data for the set of differentially expressed genes. Mean expression values for each gene in each sample type were log transformed, and the data were adjusted to center the genes relative to the overall mean. A Self-Organizing Map (SOM) was generated using the default settings to organize both genes and arrays and followed by hierarchical clustering using the average linkage method.

### Whole-mount in situ hybridization

Riboprobes containing Dig-11-UTP were synthesized with T3 or T7 polymerase (Promega) from PCR product templates. Animals were fixed with formaldehyde and processed for whole-mount in situ hybridization as previously described [38, 39] using an Intavis InsituPro VS liquid handling robot. Worms for γ-irradiation experiments were fixed 3 days after a 60 Gy treatment in a JL Shepherd Mark I Cesium-137 irradiator; control and irradiated samples were processed side-by-side through fixation and hybridization and were developed for the same length of time.

### Antibody staining

Worms stained with anti-Arrestin (1:10,000 VC-1 mAb, a kind gift from Kiyokazu Agata) were fixed with formaldehyde using the same protocol as for in situ hybridization. Those stained with anti-phospho-Histone H3 (Serine 10) (1:1000 D2C8, Cell Signaling,) were fixed with Carnoy’s solution. Following fixation, staining was carried out as previously described [39].

### RNA interference

Planarians were fed bacteria induced to express double-stranded RNA (dsRNA) against the gene of interest as previously described [39, 40]. Bacterially expressed *gfp* dsRNA was used as a negative control. For the RNAi screen, feedings were administered twice a week for three or six feedings, and animals were amputated pre-pharyngeally on the day after the last feeding, allowed to regenerate for 14 days, and then fixed for staining. The number of feedings varied in other experiments as described in the Results.

### Identification and phylogenetic analysis of Importin-α proteins

To identify planarian homologs of Importin-α proteins, Importin-α sequences from species listed in Fig. 4 were obtained from the UniProt database [41] and used for tblastn searches against the planarian genome [42] (e-value ≤ 10^−4^). Conserved protein domains, including the Importin-β binding domain and armadillo repeats were identified using PROSITE [43]. Alignment of the amino acid sequences was performed using ClustalW within MEGA5 software [44] using the default settings. The phylogenetic tree was constructed from this alignment by the Neighbor-joining method in MEGA5 using the Jones-Taylor-Thornton substitution model and settings for uniform mutation rates and pairwise gap deletion. The reliability of the tree was tested by the bootstrap method with 1000 replicates.

### Imaging

A Leica M205C microscope outfitted with Leica DFC290 camera was used to image live animals and those stained by colorimetric in situ hybridization. Fluorescent samples were imaged with a Zeiss Axiocam MRm camera mounted on a Zeiss SteREO Lumar.V12 stereomicroscope or a Zeiss Axio Observer.Z1 inverted microscope outfitted with an ApoTome for optical sectioning.

### Cell counting

The number of PH3 positive cells was counted by hand from a single focal plane image of the whole worm and normalized to the area inside a hand-drawn outline of the worm calculated by Axiozoom. Counts were performed using ImageJ 4 software [45].

### Database creation

A relational database was developed using MySQL to house multi-format data, and a web interface was created to intuitively interact with the database. The database includes a customizable BLAST search (blastn, tblastx), export and search functions, and image-viewing capability. Each record is uniquely identified by the EST contig name. Annotation (BLAST2GO, GO, KOG), expression, and phenotype data are searchable via the web interface, and search results can be downloaded into a comma-separated value file (.csv) [46].

## Results

### Changes in gene expression during head repair and regeneration

As a first step in our screen, we measured changes in gene expression on days two, three, and four of regeneration following amputation of one side of the head. We chose to focus on the head and these time points because this is a region and period where many different cell and tissue types differentiate and pattern relative to one another; we were also interested in studying the regeneration and patterning of the CNS. The nascent CNS can be visualized with the neural marker anti-Synapsin by day three of regeneration and has connected to the cephalic ganglion on the non-amputated side of the head by day four (Fig. 1a). Removing only one side of the head allowed us to examine mechanisms of repair in addition to those required for de novo regeneration of the entire head; we also aimed to identify molecular interactions between the blastema and the non-regenerating tissue that was left behind on the opposite side. Figure 1b outlines our sample collection procedure. We amputated half of the head, saved the tissue that was removed as the non-regenerating control sample, and then collected samples from the blastema and “opposite side” on days two through four of regeneration. We extracted RNA from each of these samples, reverse transcribed the RNA to cDNA and submitted these samples to the GeneChip Microarray Core at University of California San Diego for labeling and hybridization to custom Nimblegen microarrays [33, 34]. Analysis of the resulting data revealed 637 genes with significant (p ≤ 0.05) changes in gene expression in one or more of the blastema or opposite side samples compared to control (Additional files 1 and 2). Of these, 420 genes were upregulated in the blastema and 179 were downregulated. For the majority of the upregulated genes, expression peaked on day two and then declined over the rest of the time course, but a cluster of 74 genes remained highly upregulated throughout (Fig. 1c). On the opposite side of the head, we found 23 genes with increased expression and 33 with decreased expression; for 38 of these genes, the change in expression was unique to the opposite side tissue and not mirrored in the blastema.

To begin characterizing the differentially expressed genes, we grouped them into functional categories based on homology using Eukaryotic Clusters of Orthologous Groups (KOGs) (Fig. 1d). Approximately half of the genes (332/637) could be definitively assigned to a KOG group, and the others were unclassifiable due to low sequence conservation. The genes upregulated in the blastema were divided across many functional groups. The most highly represented category was “Posttranslational modification, protein turnover, chaperones”, which included homologs of MEPRIN A metalloproteinase and other proteases, proteasome subunits, and heat shock proteins. The category of “Replication, recombination and repair” was the next most common and contained several DNA replication licensing factors (MCM2, 3, 4, 5 and 7), exo- and endonucleases involved in DNA repair, and cell cycle protein CDC45, among others; representation of genes in this category is likely due to the inclusion of some postblastema tissue in our samples. Notably, the “Transcription” category, which was of interest to us because it contains proteins such as transcription factors and chromatin regulators likely to play important roles in controlling changes in gene expression during regeneration, was also highly represented among the differentially expressed genes. Nearly half (28 out of 62) of the annotated downregulated genes and almost as many upregulated genes were identified as functioning in “Signal transduction mechanisms”. Several genes potentially involved in neural function were among those downregulated in the signal transduction class, including homologs of three human GABA receptors (GBRB2, GBRB3, and GABRR2) and a sodium-dependent noradrenaline transporter, SLC6A2.

In samples from the side of the head opposite to the blastema of regenerates, differentially-expressed genes again fell into diverse functional categories. Genes upregulated in the opposite side tissue but not in the blastema included a homolog of KCNN3, a small conductance calcium-activated potassium channel with neuronal function in humans. “Signal transduction” was again the most highly represented functional category among the downregulated genes and included Wnt signaling protein Frizzled and homologs of an acetylcholine receptor and a serine/threonine kinase. Given that most cell types in the animal are contained within the samples we collected, we expected to find a broad range of functional categories represented in our expression data.

### Identification of genes expressed in the blastema, neoblasts, and CNS

**Fig. 2:**
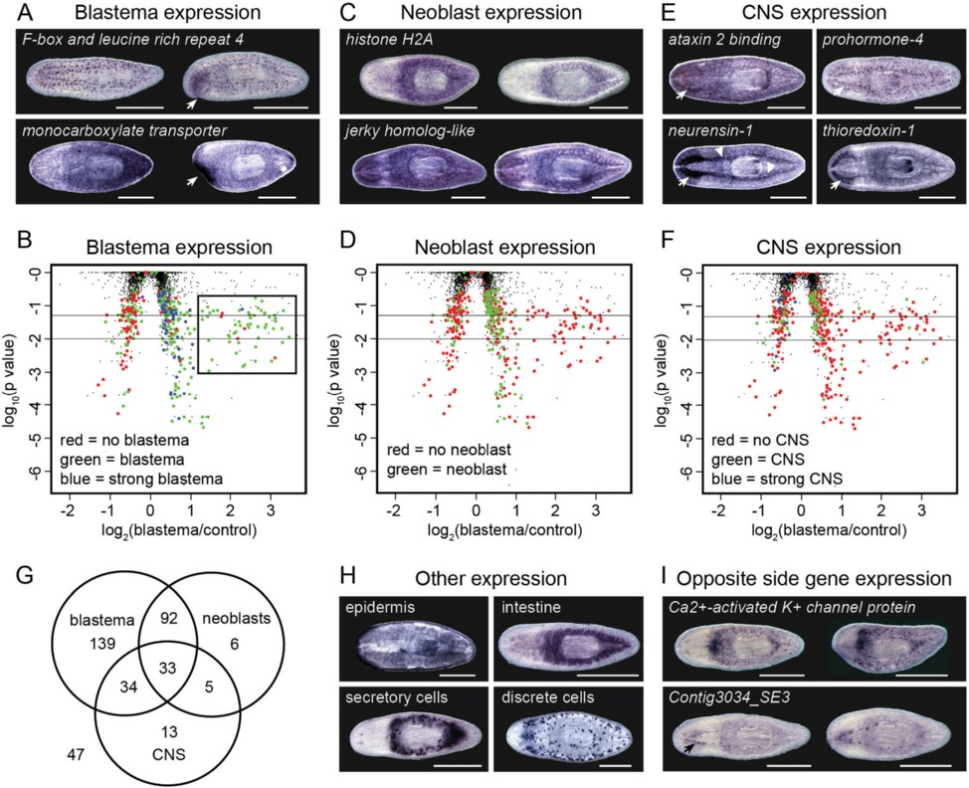
Identification of genes expressed in tissues of interest and validation of microarray results. Representative examples from the WISH screen. **a** Blastema expression. The lower half of the head of the animal on the right in each pair was removed two to four days before fixation, on the day of greatest expression change for each gene. Arrows indicate the blastema. **b** Volcano plot of p-value vs. fold change for blastema to control comparison colored to indicate genes with no increased expression in the blastema (*red*), moderate blastema expression (*green*; e.g., *F-box and leucine rich repeat 4*) or strong blastema expression (*blue*; e.g., *monocarboxylate transporter*). **c** Neoblast expression. The animals on the right were irradiated (60 Gy) 3 days before staining to destroy the neoblasts. The animals on the left are non-irradiated. **d** Volcano plot colored to indicate neoblast expression; green marks genes detected in neoblasts and red marks non-neoblast genes. Genes were scored as expressed in neoblasts if staining was reduced following irradiation, as for *histone h2a* and *jerky homolog-like*. **e** CNS expression. Arrows and arrowheads indicate expression in the cephalic ganglia and ventral nerve cords, respectively. **f** Volcano plot colored to indicate genes with no CNS expression (*red*), weak CNS expression (*green*; e.g., *Rb-like protein*) or strong CNS expression (*blue*; e.g., *prohormone-4*, *neurensin-1*, and *thioredoxin*). **g** Venn diagram showing overlap of expression between tissues of interest. **h** Examples of genes not expressed in the blastema, neoblasts, or CNS, but in the epidermis (*fam166b protein*), intestine (*Contig3907_SE3*), secretory cells (*Contig7601_SE3*), or other discrete cells (*mannosyl phosphorylinositol ceramide synthase sur1)*. **i** Examples of WISH to genes with proposed opposite side of the head expression. The lower half of the head of the animal on the right in each pair was amputated 2 to 4 days before fixation. The arrow indicates cephalic ganglia expression. Animals were imaged ventrally, anterior to the left. Scale = 0.5 mm

Based on our expectation that genes involved in head regeneration and repair would be expressed in the blastema, neoblasts, or CNS, we further analyzed the genes identified from our microarray study by whole-mount in situ hybridization (WISH). We performed these experiments to determine which cell and tissue types they are expressed in and to validate our microarray results. Additional file 3 provides a list of the genes tested and a summary of the results. Among 260 genes that were identified as upregulated in the blastema by our microarray analysis, we found 243 that were expressed at a higher level in the blastema than in the non-regenerating tissue by WISH. For some, the increased expression was tightly confined to the blastema itself (e.g., *F-box and leucine rich repeat 4*), whereas others were strongly expressed both in the blastema and in the area beneath it (e.g., *monocarboxylate transporter*) (Fig. 2a). Sixty genes appeared strongly upregulated in the blastema based on in situ hybridization, but surprisingly only one of these fell within the group of genes showing the largest fold-change by microarray (boxed region in Fig. 2b). This can be explained by noting that in non-regenerating animals, many of the genes with the largest fold-change were detected almost exclusively in the secretory cells surrounding the pharynx - any expression in the blastema represented a large change compared to the control head region, even if the resulting expression was not particularly strong. Taken together, the in situ staining validated the microarray results with regard to blastema expression.

To identify genes expressed in the neoblasts, we performed WISH in control animals and planarians treated with γ-irradiation to destroy the neoblasts [5, 47, 48]. We found 139 genes with reduced staining in the irradiated samples. Some of these genes were expressed only in the neoblasts (e.g., *histone H2A*), whereas others were also strongly expressed in other tissues (e.g., *jerky homolog-like*), including the CNS and intestine (Fig. 2c). The vast majority of neoblast-expressed genes were also expressed in the blastema (127 out of 139 genes; Fig. 2g), and many were found among the genes identified by microarray to be upregulated in the blastema (Fig. 2d).

We identified 81 genes expressed in the CNS exclusively or in combination with other tissues. Some genes were expressed in the CNS at levels similar to their expression in other tissues and were classified as simply having CNS expression, whereas others were more highly expressed in the CNS and were classified as “strong CNS” genes (Fig. 2e and f). Genes with strong CNS expression were enriched among those that appeared downregulated in blastema samples compared to control on the microarrays (Fig. 2f). This is likely not due to actual downregulation but more representative of genes that are highly expressed in the cephalic ganglia of intact animals and that are not strongly upregulated in the blastema. Among the genes with strong CNS expression, some appeared pan-neuronal (e.g., *neurensin-1*) whereas others were expressed in a subset of neuronal cells or tissues (e.g., *prohormone-4* and *thioredoxin*) (Fig. 2e). Besides genes expressed in the blastema, neoblasts and/or the CNS, 52 genes were found primarily in other tissues, including the epidermis, intestine, secretory cells, or other discrete cells (Fig. 2h).

We also performed in situ hybridization to 19 genes that were identified by microarray to have changed in expression only on the opposite side of the head (versus also changing in the blastema) during regeneration. Of these, seven were identified as potentially upregulated in the opposite side tissue. We did not observe an obvious increase in expression in the opposite side of the head over that of the surrounding tissue, although there may have been a more widespread increase in expression that included this region. The staining pattern for several of these genes (5 of 7) was similar to that of the *Ca*^*2+*^*activated K*^*+*^*channel* shown in Fig. 2i, with expression mainly in the mesenchyme. None of these seven genes were expressed in the CNS. In contrast, the majority of genes (7 of 12) identified as downregulated in the opposite side tissue showed strong CNS expression, as exemplified by Contig3034_SE3 (Fig. 2i).

In total, we characterized the expression patterns of 390 genes, most of which had not been previously reported. We have created a publicly accessible database to house our in situ data. Images of the expression patterns of all genes analyzed in our screen are available at http://planaria.sdsu.edu.

### A targeted RNAi screen to identify genes required for regeneration

**Fig. 3:**
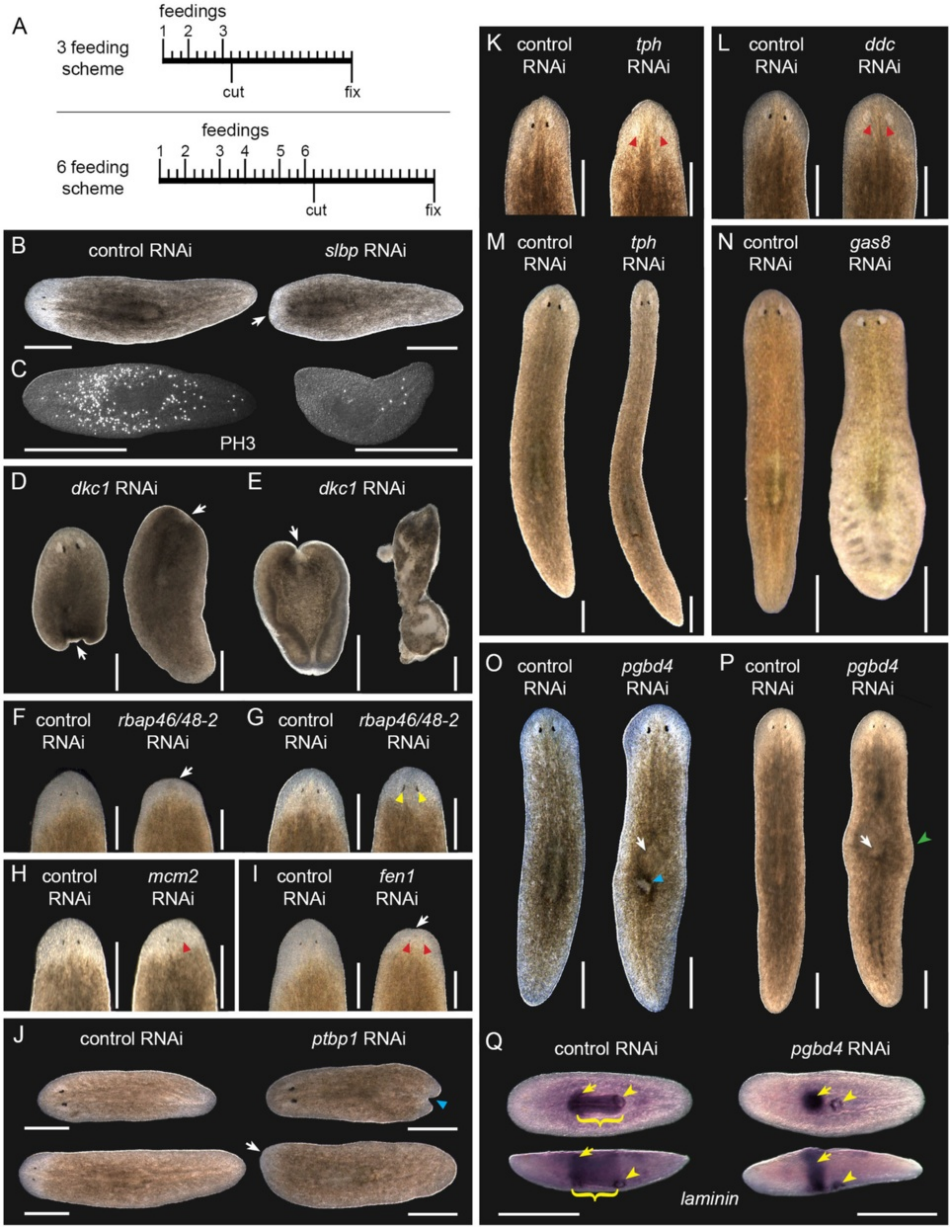
Representative images of phenotypes observed following RNAi knockdown. **a** RNAi feeding schedules. Phenotypes in panels (**b**), (**c**), and (**g**) appeared after three feedings, panel (**l**) after 12 feedings, and the remainder after six feedings. **b**-**c** Loss of neoblasts in *Smed-slbp(RNAi)*. Arrow indicates reduced-size blastema. Anti-PH3 labels the mitotic neoblasts. Live animals were imaged on day seven of regeneration, and stained animals were fixed on day 14. **d**-**e** Phenotypes of *Smed-dkc1(RNAi)*. Arrows indicate missing blastemas. **d** = day seven following pre-pharyngeal amputation, **e** = ventral curling and lysis on day 14. **f** Impaired regeneration following six *Smed-rbap46/48-*2 dsRNA feedings. Animals imaged on day seven. **g** Abnormally elongated photoreceptor pigment (*yellow triangles*) in day seven regenerates following three feedings against *Smed-rbap46/48-2*. **h** Asymmetric photoreceptors of *Smed-mcm2(RNAi)*. The red triangle indicates underdeveloped photoreceptor in day six regenerates. **i** Reduced blastema (*arrow*) and underdeveloped photoreceptors (*red triangles*) in day six *Smed-fen1(RNAi)* regenerates. **j** Reduced blastema (*arrow*) and forked tail (*blue triangle*) in day seven *Smed-ptbp1(RNAi)* regenerates. **k** *Smed-tph(RNAi)* animals lacking photoreceptor pigment on day 14 of regeneration. Red triangles indicate missing pigment. **l** Reduction of photoreceptor pigment in *Smed-ddc(RNAi)*. Red triangles indicate photoreceptor pigment cups. **m** Elongated body shape following extended *Smed-tph* RNAi. **n** Edema following *Smed-gas8* RNAi. The animal shown is affected in the posterior. **o**-**q** *Smed-pgbd4(RNAi)* phenotypes. White arrows indicate dorsal humps in uninjured animals (**o**) and day 14 regenerates (**p**). The blue triangle indicates a post-pharyngeal lesion, and the green arrowhead indicates lateral bulging. **q** WISH to pharynx marker *laminin* in *Smed-pgbd4(RNAi)*. Upper animals are shown ventrally, and lower animals were imaged from the side, with dorsal toward the top. Anterior is to the left. Yellow arrows and arrowheads indicate the base of the pharynx and its ventral opening, respectively. Brackets indicate the pharynx in controls. Scale = 0.5 mm for all panels

We chose 156 genes from those differentially expressed during head regeneration to knock down by RNAi (Additional file 4). We gave higher priority to genes that were expressed in tissues of interest (blastema, neoblasts, CNS) from our WISH experiments or that we hypothesized would function in head/CNS regeneration based on their homology. Planarians were fed double-stranded RNA (dsRNA) twice per week for either three or six feedings and then amputated anterior to the pharynx and observed through 14 days of regeneration (Fig. 3a). Knockdown of 25 genes resulted in phenotypes (Table 1), which included stem cell loss, impaired regeneration, effects on patterning or differentiation, and defects affecting specific tissues such as the photoreceptors and pharynx. The results for genes in each of the phenotype categories are described below.

**Table 1:** Summary of 25 genes producing RNAi phenotypes

Gene	E–value	KOG group	Functional category	Phenotype
*Smed–slbp*	1.00E–10	Histone mRNA stem–loop binding protein	[A] RNA processing and modification	Loss of stem cells
*Smed–sart3*	4.00E–102	RNA–binding protein SART3	[A] RNA processing and modification	Loss of stem cells
*Smed–smarcc–1*	2.00E–08	Chromatin remodeling factor subunit and related transcription factors	[B] Chromatin structure and dynamics	Loss of stem cells
*Smed–cycD*	2.00E–10	G1/S–specific cyclin D	[D] Cell cycle control, cell division, chromosome partitioning	Loss of stem cells
*Smed–espl1*	3.00E–32	Regulator of spindle pole body duplication	[D] Cell cycle control, cell division, chromosome partitioning	Loss of stem cells
*Smed–rrm2b–1*	6.00E–28	Ribonucleotide reductase, beta subunit	[F] Nucleotide transport and metabolism	Loss of stem cells
*Smed–rrm2b–2*	3.00E–143	Ribonucleotide reductase, beta subunit	[F] Nucleotide transport and metabolism	Loss of stem cells^*^
*Smed–pdss2/dlp1*	1.00E–28	Geranylgeranyl pyrophosphate synthase/ Polyprenyl synthetase	[H] Coenzyme transport and metabolism	Loss of stem cells^*^
*Smed–dkc1*	5.00E–149	Pseudouridine synthase	[J] Translation, ribosomal structure and biogenesis	Loss of stem cells
*Smed–emg1/nep1*	1.00E–26	Protein required for 18S rRNA maturation and 40S ribosome biogenesis	[J] Translation, ribosomal structure and biogenesis	Loss of stem cells^*^
*Smed–lig1*	9.00E–125	ATP–dependent DNA ligase I	[L] Replication, recombination and repair	Loss of stem cells
*Smed–prim2*	1.00E–99	Eukaryotic–type DNA primase, large subunit	[L] Replication, recombination and repair	Loss of stem cells
*Smed–mcm7*	0	DNA replication licensing factor, MCM7 component	[L] Replication, recombination and repair	Loss of stem cells^*^
*PL08006B2E08*	––	No significant homology	none	Loss of stem cells
*Smed–rbap46/48–2*	3.00E–79	Nucleosome remodeling factor, subunit CAF1/NURF55/MSI1	[B] Chromatin structure and dynamics	Six feedings – reduced/delayed regeneration, reduced mitosis Three feedings – elongated photoreceptor pigment
*Smed–mcm2*	2.00E–148	DNA replication licensing factor, MCM2 component	[L] Replication, recombination and repair	Reduced/delayed regeneration, asymmetric photoreceptors, reduced mitosis
*Smed–ptbp1*	2.00E–54	Polypyrimidine tract–binding protein	[A] RNA processing and modification	Reduced/delayed regeneration, forked tail, inching movement, lysis
*Smed–fen–1*	1.00E–149	5′–3′ exonuclease	[L] Replication, recombination and repair	Reduced/delayed regeneration
*Smed–morf4l1/mrg–1*	5E–26	Dosage compensation regulatory complex/ histone acetyltransferase complex, subunit MSL–3/MRG15/EAF3	[BK] Chromatin structure and dynamics, transcription	Reduced/delayed regeneration, lysis
*Smed–ddc*	2.00E–57	Aromatic–L–amino–acid/L–histidine decarboxylase	[E] Amino acid transport and metabolism	Faint photoreceptor pigment
*Smed–tph*	0	Aromatic amino acid hydroxylase	[E] Amino acid transport and metabolism	No photoreceptor pigment, elongated, inching movement
*Smed–gas8*	9.00E–87	No significant homology	None	Edema
*Smed–pgbd4*	1.00E–05	No significant homology	None	Lesion at posterior of pharynx, dorsal hump, bulged sides, loss of pharynx, impaired photoreceptor development
*Smed–b9d2*	9.00E–75	Uncharacterized conserved protein	[S] Function unknown	Inching movement
*Smed–ima–1*	1.00E–104	Karyopherin (importin) alpha	[U] Intracellular trafficking, secretion, and vesicular transport	Collapse toward midline, cyclops/asymmetric photoreceptors, reduced mitosis

Only genes producing a phenotype are shown; see Additional file 4 for a full list of genes tested. Gene names were assigned based on homology from BLASTx searches against the NCBI database. E–values are the lower value between BLASTx of the cloned EST or a longer sequence from published transcriptomes [13, 14] against the corresponding human protein. KOG group and functional category assignments were made using the eukaryotic Clusters of Orthologous Groups database [49]. An asterisk (*) indicates that loss of stem cells was verified by staining with anti–phospho–Histone H3

### Differentially expressed genes required for neoblast survival/maintenance

Knockdown of 14 genes resulted in phenotypes associated with loss of the stem cells, which include impaired blastema formation, head regression, lesions, ventral curling and lysis (Table 1 and Fig. 3b, d and e). Staining with anti-phospho-Histone H3 (PH3) revealed a striking reduction in the number of mitotic cells relative to controls following RNAi against four of the genes that had the “loss of stem cells” phenotype (Fig. 3c, marked in Table 1); staining was not performed for the other genes in this group, in most cases due to death of the animals by lysis prior to the end of the two week observation period. All 14 of the genes in this phenotypic class were upregulated in the blastema based on both microarray and WISH data, and 12 of them (all except *Smed-smarcc-1* and *PL08006B2E08*) were expressed in the stem cells. These data suggest that these genes play important roles in the survival or maintenance of planarian stem cells.

### Genes required for blastema formation and regenerative capacity

Knockdown of five other genes resulted in a general reduction in regeneration ability without the dramatic loss of stem cells observed in the previous group. For each of these genes, blastemas developed but were smaller than those in controls, and photoreceptors were delayed in formation or underdeveloped relative to controls at the same time point (Fig. 3f-j). There was a three- to four-fold decrease in the number of mitotic cells following knockdown of *Smed-rbap46/48-2* (to 75 ± 24 cells/mm^2^ vs. 267 ± 70 cells/mm^2^ for *gfp(RNAi)*, *n* = 8 animals/gene) and *Smed-mcm2* (to 69 ± 28 cells/mm^2^ vs. 292 ± 70 cells/mm^2^ for *gfp(RNAi)*, *n* = 8 animals/gene).

The impaired regeneration phenotype was accompanied by additional defects in *Smed-rbap46/48-2(RNAi), Smed-mcm2(RNAi)* and *Smed-ptbp1(RNAi)* animals. In contrast to the underdeveloped photoreceptors observed following six dsRNA feedings targeting *Smed-rbap46/48-2*, shortening the treatment to three feedings uncovered a defect in photoreceptor morphology in which the pigment cups appeared abnormally elongated at early stages of regeneration (Fig. 3g). In some *Smed-mcm2(RNAi)* animals (*n* = 9/30 tails after six RNAi feedings), the photoreceptors were asymmetric in size or only one photoreceptor fully developed (Fig. 3h). Finally, following knockdown of *Smed-ptbp1*, head fragments regenerating a new tail sometimes developed a forked blastema with two separate points rather than the typical tapered tail (14/30 heads after 6 RNAi feedings; Fig. 3j), and both head and trunk fragments displayed abnormal inching movements. The knockdown results for this group of genes as a whole suggest they may act in cell differentiation and/or tissue patterning during regeneration.

### Other defects and effects on specific tissues or cell types

Knockdown of five other genes resulted in phenotypes distinct from those described above in that they did not affect general stem cell function or blastema formation but instead had more specific effects. Two of these genes, *dopa decarboxylase* (*Smed-ddc*) and *tryptophan 5-hydroxylase* (*Smed-tph*), were required for production of photoreceptor pigment; in trunks regenerating their heads, *Smed-tph(RNAi)* animals lacked photoreceptor pigment entirely (Fig. 3k), and the pigment cups in *Smed-ddc(RNAi)* animals were visible as faint brown spots rather than the usual dark black color (Fig. 3l). Both *Smed-tph* and *Smed-ddc* are expressed in the pigment cup (Additional file 5) [20]. In addition to the photoreceptor phenotype described above, extended knockdown of *Smed-tph* caused the worms to become longer and thinner (Fig. 3l). The length-to-width ratio of non-regenerating *Smed-tph(RNAi)* animals after 12 dsRNA feedings was 8.4 ± 1.2 compared to 5.6 ± 0.6 for *gfp(RNAi)* controls (students’ *t*-test p-value < 0.0001). These longer *Smed-tph(RNAi)* animals also displayed abnormal inchworm-like movement rather than the usual gliding.

RNAi against *growth arrest-specific protein 8* (*Smed-gas8*) led to edema (Fig. 3n), a phenotype typically associated with dysfunction of the protonephridia. Finally, we observed movement defects (inching) following knockdown of a homolog of *B9 domain-containing protein 2* (*Smed-b9d2*). This phenotype was most noticeable shortly after the final dsRNA feeding in both uninjured and regenerating animals and seemed to wear off over time after the RNAi feedings stopped.

Knockdown of a protein with weak homology to *PiggyBac transposable element-derived protein 4* (*Smed-pgbd4*) led to the formation of a raised hump over the pharynx and a single lesion at the posterior end of the pharynx on the dorsal side of the animal (Fig. 3o). In some (9/50 trunks after six RNAi feedings) regenerating animals, we also observed lateral bulges near the pharynx (Fig. 3p). Staining with the pharynx marker laminin in *Smed-pgbd4(RNAi)* trunk fragments after 14 days regeneration or in uninjured animals following a period of 10 days starvation after the final dsRNA feeding revealed that the pharynx was lost after this treatment (Fig. 3q).

The observation that knockdown of genes in this group leads to specific defects rather than a general loss of regenerative capacity suggests that their products may act in pathways that direct regeneration or maintenance of particular tissues or cell types, including the photoreceptors, ciliated cells and the pharynx.

### Planarians have two homologs of *importin-α* with distinct expression patterns and function

**Fig. 4:**
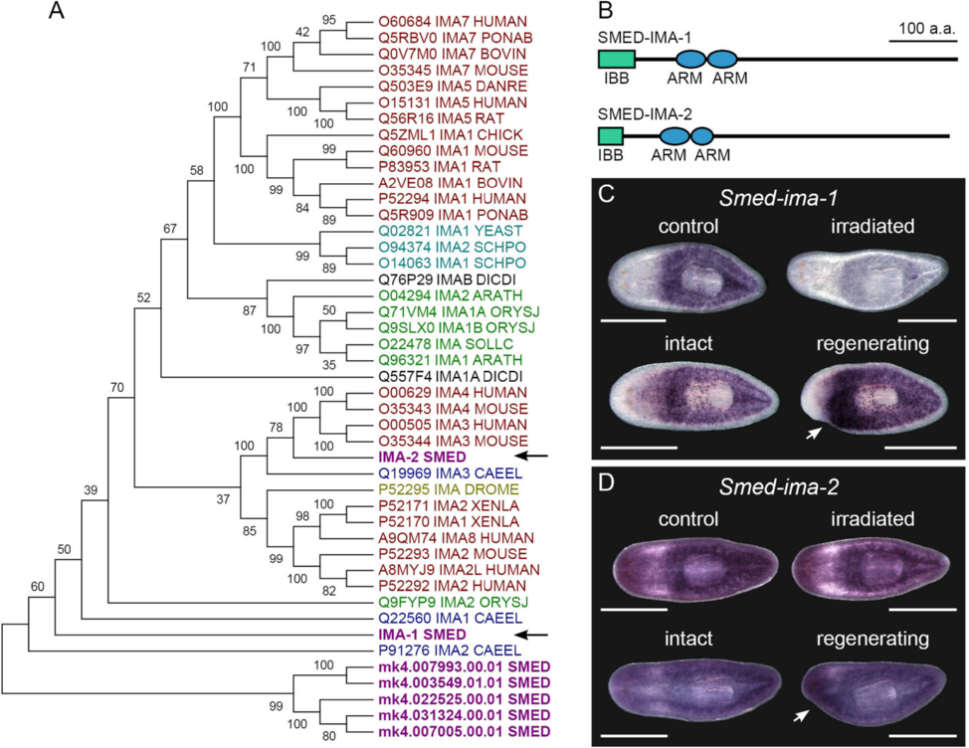
Analysis of *importin-α* homologs in *S. mediterranea*. **a** Phylogenetic analysis of Importin-α (ΙΜΑ) homologs. Each protein is labeled with Uniprot accession number, gene name, and species. Species abbreviations are as follows: HUMAN = *Homo sapiens*, PONAB = *Pongo abelii*, BOVIN = *Bos taurus*, MOUSE = *Mus musculus*, DANRE = *Danio rerio*, RAT = *Rattus norvegicus*, CHICK = *Gallus*, YEAST = *Saccharomyces cerevisiae*, SCHPO = *Schizosaccharomyces pombe*, DICDI = *Dictyostelium discoideum*, ARATH = *Arabidopsis thaliana*, ORYSJ = *Oryza sativa* subspecies *japonica*, SOLLC = *Solanum lycopersicum*, SMED = *Schmidtea mediterranea*, CAEEL = *Caenorhabditis elegans*, DROME = *Drosophila melanogaster*, XENLA = *Xenopus laevis*. Arrows indicate *Smed-ima-1* and *Smed-ima-2*. **b** Domain structure of *Schmidtea mediterranea* Importin-α proteins. IBB = Importin-β binding domain. ARM = Armadillo repeats. **c**-**d** Whole-mount in situ hybridization to *Smed-ima-1* and *Smed-ima-2*. The irradiated worms were treated with 60 Gy γ-irradiation 3 days prior to fixation to destroy the stem cells. The regenerating animals were amputated to remove the half of the head oriented toward the bottom of the picture two days prior to fixation. Arrows indicate the blastema. Animals were imaged from the ventral side with anterior to the left. Scale = 0.5 mm

The final gene that produced a phenotype upon knockdown in our screen was a homolog of the nuclear transport factor Importin-α that we named *Smed-ima-1*. Knockdown of this gene led to defects in differentiation and patterning of regenerating tissues along with a decrease in the number of mitotic cells (described in more detail later). Importin-α homologs in other species aid in the transport of NLS-containing proteins into the nucleus by acting as an adaptor between the target proteins and Importin-β, which in turn interacts with the nuclear pore complex [50]. Intrigued by the possibility that regulated nuclear import of proteins could be important for planarian regeneration, we decided to characterize the Importin-α gene family in *S. mediterranea*. In addition to *Smed-ima-1*, we identified another member of this family in the *S. mediterranea* genome, which we named *Smed-ima-2* (Fig. 4a). Each of these proteins contains two Armadillo repeats, which may mediate protein-protein interactions, and an Importin-β binding domain (IBB), which is required for the interaction between Importin-α and Importin-β (Fig. 4b). A handful of other transcripts (labeled in the phylogenetic tree by the mk4 names given to them by the Maker gene prediction program in SmedGD [51]) shared some sequence similarity with this family but did not contain the Importin-β binding domain (Fig. 4b) and could not be detected by in situ hybridization, suggesting that they are not functional homologs. In contrast to *Smed-ima-1*, which was predominantly expressed in the stem cell population (Fig. 4c), *Smed-ima-2* was ubiquitously expressed but still present in the stem cells based on its reduction following γ-irradiation (Fig. 4d). *Smed-ima-1* was strongly upregulated in the region beneath the blastema during regeneration.

**Fig. 5:**
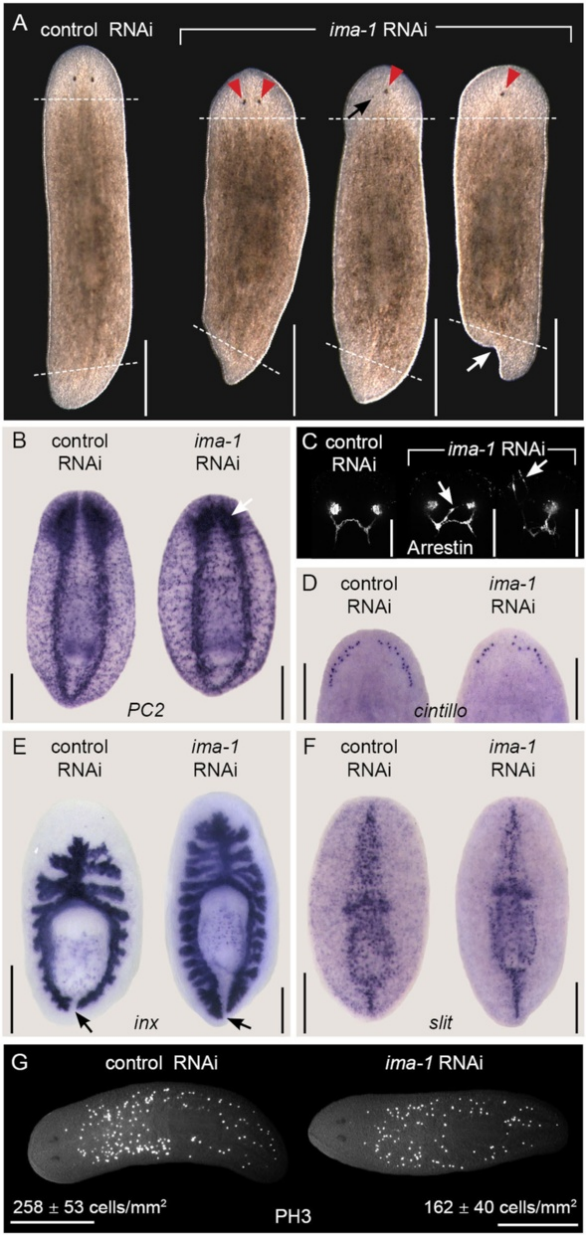
*Smed-ima-1* is required for normal stem cell function and regeneration. **a**-**f** Images of control and *Smed-ima-1(RNAi)* animals fed bacterially expressed dsRNA targeting each gene four times over 2 weeks then amputated transversely both pre- and post-pharyngeally. Animals were imaged or fixed on day 10 of regeneration. Anterior is toward the top. Live animals were imaged from the dorsal side and all others were imaged ventrally. Scale bars = 0.5 mm for (**a**) and (**g**), 0.25 mm for (**b**) and (**d**-**f**), 0.1 mm for (**c**). **a** Live animals following RNAi treatment. Dashed lines indicate amputation sites. Red triangles indicate photoreceptors forming abnormally close to the midline, and the black arrow indicates a mis-positioned and underdeveloped photoreceptor. The white arrow indicates forking of the tail blastema. **b** In situ hybridization to the neuronal marker *Smed-pc2*. White arrow indicates small cephalic ganglia collapsed toward the midline. **c** Staining with anti-Arrestin antibody (Arrestin) to mark photoreceptor neurons. Arrows indicate aberrant neuronal projections. **d** In situ hybridization to *Smed-cintillo*, which labels sensory neurons. **e** In situ hybridization to *Smed-inx* to label the intestine. Arrows mark space between the two posterior intestinal branches. **f** In situ hybridization to midline marker *Smed-slit*. **g** Uninjured animals stained with anti-phospho-Histone H3 antibody (PH3) to mark mitotic cells following six dsRNA feedings. Anterior is to the left

The two Importin-α homologs had distinct functions based on RNAi knockdown experiments. Knockdown of *Smed-ima-2* caused a rapid loss of stem cells accompanied by ventral curing and lysis (data not shown; also observed by Reddien et al. [48]), suggesting that *Smed-ima-2* may serve an essential function in bulk nuclear import of NLS-containing proteins. The *Smed-ima-1* RNAi phenotype was more specific; when animals were fed *Smed-ima-1* dsRNA four times over two weeks then amputated pre-pharangeally, the photoreceptors often appeared closer together than in *gfp(RNAi)* controls (*n* = 21/113) (Fig. 5a). In other animals, one photoreceptor was very small or absent (*n* = 23/113), or a single cycloptic photoreceptor appeared near the center of the head (*n* = 32/113) (Fig. 5a). The abnormality of the photoreceptors extended to their neuronal connections (*n* = 4/4), which we visualized by staining RNAi-treated animals with an antibody against Arrestin [52] (Fig. 5c). The cephalic ganglia in *Smed-ima-1(RNAi)* planarians were reduced in size, with an average area of 0.114 ± 0.015 mm^2^/mm animal length compared to 0.145 ± 0.019 mm^2^/mm animal length in controls (*n* = 4-5/group, Students’ *t*-test p-value = 0.031) (Additional file 6A). *Smed-ima-1(RNAi)* cephalic ganglia also appeared less developed than in controls and were also collapsed toward the midline (*n* = 5/5) (Fig. 5b). The smaller size of the cephalic ganglia may have been due to a defect in differentiation; this defect is further illustrated by a reduction in the number of regenerated sensory neurons, from 28.5 ± 1.2 *cintillo* positive cells/mm length in control to 18.9 ± 2.7 in *Smed-ima-1(RNAi)* (*n* = 5/group, Students’ *t*-test p-value < 0.0001) (Fig. 5d and Additional file 6B). The regenerating tails of trunk fragments also developed abnormally in many cases, with the blastema growing asymmetrically slanted or forked (*n* = 24/64) (Fig. 5a). Interestingly, we did not observe similar defects in the tail blastema morphology of regenerating head fragments.

The abnormal positioning of the photoreceptors and cephalic ganglia with respect to the midline led us to investigate whether *Smed-ima-1(RNAi)* caused other defects in midline patterning. Knockdown of *Smed-slit*, a known regulator of midline patterning, leads to fusion of the two posterior branches of the intestine during regeneration [53], however knockdown of *Smed-ima-1* did not lead to the same phenotype (Fig. 5e). We also assayed *Smed-slit* mRNA expression by WISH in *Smed-ima-1* knockdown animals and did not find any overt difference in its pattern or levels (Fig. 5f). Therefore, the midline collapse phenotype of *Smed-ima-1* RNAi does not appear to be caused by a defect in *Smed-slit* regulation.

We also observed a reduction in the number of mitotic cells in *Smed-ima-1(RNAi)* animals compared to controls. After six feedings of dsRNA over three weeks, the number of PH3^+^ cells in intact animals was reduced from 258 ± 53 cells/mm^2^ in *gfp(RNAi)* controls to 162 ± 40 cells/mm^2^ in *Smed-ima-1(RNAi)* (*n* = 15–16 animals/gene, Students’ *t*-test p-value < 0.0001) (Fig. 5g). Worms treated with dsRNA against *Smed-ima-1* for extended periods of time (more than 4 weeks) began to show other signs of stem cell dysfunction, including head regression and lysis. The RNAi phenotypes of the two *Smed-importin*-*α* genes suggest that regulated nuclear import of proteins is a key factor in stem cell function.

Our functional genomics screen identified several previously uncharacterized genes required for neoblast maintenance and blastema formation and others affecting regeneration or homeostatic maintenance of specific cell and tissue types. This, along with our implication of a nuclear import factor in patterning of regenerating tissues provides new insights into the molecular basis of planarian regeneration.

## Discussion

We performed a screen to identify genes required for stem cell-based tissue replacement, approaching the problem from multiple angles. First, we identified genes differentially expressed during regeneration of one half of the head. We removed only one side of the head in order to look for genes required not only for de novo head regeneration, but also for those involved in repair and reconnection to tissue that remained following injury. We examined expression over a time course from two to four days after amputation to go beyond the initial wound response to include the period when patterning and reconnection of tissues such as the CNS occur. Working from the list of differentially expressed genes, we identified genes expressed in tissues of interest to us (neoblasts, blastema, and CNS) and then examined the function of selected genes by RNAi.

In the course of the screen, we identified genes involved in all aspects of regeneration, from neoblast maintenance to differentiation and patterning. Many of the genes required for neoblast maintenance are genes with essential cellular functions, such as ribosome biogenesis and DNA replication. Others, however, may play a more specific role in cell cycle regulation or maintaining pluripotency. For example, SART3 (Squamous cell carcinoma antigen recognized by T-cells 3), an RNA binding nuclear protein involved in pre-mRNA splicing [54], tumor rejection [55], and HIV-1 replication [56], has also been implicated in maintaining pluripotency of human embryonic stem cells [57]. In addition, *Smed-smarcc-1*, which Wenemoser et al. [18] identified as upregulated as part of the initial wound response, is predicted based on homology to human *SMARCC2* to play a role in chromatin-mediated regulation of gene expression. SMARCC2 is part of the npBAF and nBAF chromatin remodeling complexes, which are required for proliferation of neural progenitors and dendrite growth in differentiated neurons, respectively [58]. The loss of neoblasts phenotype in *Smed-smarcc-1(RNAi)* animals suggests it may act in a broader capacity in maintaining pluripotency and mitotic activity of planarian neoblasts.

The phenotypes we observed for other genes in the screen were also consistent with their putative function based on homology. For example, *ddc* is required in other species for synthesis of melanin [59], the primary pigment of the planarian photoreceptor [60]. *Smed-ddc* is expressed in the photoreceptors (Additional file 5 and [20]), and knockdown leads to a reduction in photoreceptor pigment, consistent with a function in melanin production. RNAi against *Smed-tph* led to loss of photoreceptor pigment and changes in body morphology and movement, with animals becoming longer and thinner over an extended feeding scheme and losing their ability to glide smoothly. Our observation that *Smed-tph* is involved in pigmentation is consistent with the reported role of *Smed-tph* in eye melanogenesis [27]. Additionally, because *Smed-tph* is required for serotonin biosynthesis [61–63], the *Smed-tph* RNAi movement defect is in line with previous observations that the coordinated wave-like motion of the ventral cilia of planarians is controlled by serotonin [9, 64]. Along these same lines, the movement defects we observed following knockdown of *Smed-b9d2* in planarians are likely directly linked to a conserved function in the production of the ventral cilia the animals use for gliding. B9D2 homologs play an important role in ciliogenesis in other species [65, 66], and loss-of-function mutations in this gene lead to Meckel syndrome in humans, which is characterized by renal cysts and other abnormalities related to cilia dysfunction [67]. Further investigation of the potential role of *Smed-b9d2* in planarian ciliogenesis could yield information useful for understanding and treating this disease.

### Communication between the blastema and non-regenerating tissue

We identified 38 genes that displayed differential expression in comparisons between samples collected from the non-regenerating side of the head during regeneration and control samples from non-regenerating animals. These genes are potentially involved in communication between the blastema and surrounding tissue to instruct the development of missing tissues in injured animals. We knocked down expression of 14 of these genes, seven of which were predicted to be upregulated and seven downregulated during regeneration based on the microarrays. Among these experiments, we only observed a phenotype following knockdown of *Smed-tph*, and this phenotype (lack of photoreceptor pigment and movement defects) was not consistent with *Smed-tph* acting as a coordinator in cellular communication between the blastema and non-regenerating tissues. Our lack of success finding any genes specifically involved in this process may have been due to our focus on only genes with a significant change in expression during regeneration; if the genes we aimed to find were already expressed in the non-regenerating animals to coordinate normal cell turnover and did not react to the onset of regeneration with a change in expression, then we would have missed them in our screen. Expanding the screen to additional genes or using other criteria (e.g., selecting genes to knock down based on homology to known signaling molecules) may eventually uncover genes involved in the communication process.

### Nuclear import as a regulatory mechanism during regeneration

Nuclear import of proteins that contain a nuclear localization signal (NLS) is facilitated by members of the karyopherin protein family, Importin-α and Importin-β. In the classical nuclear import pathway, the NLSs of target proteins are recognized and bound by Importin-α*,* which serves as an adapter between the NLS and Importin-β [68]. Transport of the cargo into the nucleus is then accomplished through interactions of Importin-β with the nuclear pore complex. Many species express several distinct Importin-α proteins with variations in binding affinity for different NLS sequences [68]. This specificity allows individual Importin-α family members to govern localization of unique sets of target proteins. The ability to grant or limit access to the nucleus to other proteins such as transcription factors allows Importin-α proteins to serve as regulatory molecules that affect cell fate [69, 70]. For example, control of the nuclear localization of transcription factor OCT3/4 by Importin-α1 affects maintenance of mouse ES cells in a pluripotent state, and control of BRN2 and SOX2 localization by Importin-α5 regulates their differentiation toward a neural fate [71]. Additionally, the observation that different Importin-α subtypes with distinct target specificities are expressed in various temporal and spatial patterns during mouse spermatogenesis has led to the proposal that regulated nuclear import plays a role in this process [72].

RNAi knockdown of the two Importin-α homologs in *Schmidtea mediterranea* led to distinct phenotypes. We found that *Smed-ima-2* is required for general stem cell survival, while *Smed-ima-1* is important for stem cell maintenance, differentiation, and patterning of regenerating tissues. We hypothesize that these proteins mediate nuclear localization of distinct groups of target proteins to achieve stem cell maintenance and regulate cellular differentiation and tissue patterning during regeneration. Interestingly, Labbé et al. previously demonstrated that knockdown of nuclear pore complex components NUP93 and NUP205 in planarians leads to defects in tissue homeostasis [13]. Together these results establish planarians as a model system for further investigation of the role of nuclear import in development, and identifying specific target proteins will be the next step toward a greater understanding of this process *in vivo*.

### Planarian in situ database

We have created an online database to make our in situ hybridization data available to the public. It contains the sequence, annotation, expression, and RNAi phenotype data, as well as links to images of in situ hybridization experiments where available. A custom BLAST page was created to allow for searches against the EST contig sequences. The database is available at http://planaria.sdsu.edu.

## Conclusions

We have identified new genes involved in various aspects of planarian regeneration and stem cell function. Most notably, characterization of *Smed-ima-1* as a key player in these processes opens the door to the study of controlled nuclear import of macromolecules as a regulatory mechanism in regeneration. Additionally, the online database we created to house our in situ data should serve as a valuable resource for the planarian research community.

### Availability of supporting data

The data sets supporting the results of this article are available in the Gene Expression Omnibus (GEO) repository, accession GSE62551, and at http://planaria.sdsu.edu/.

## Additional files

Additional file 1:
**Identifies the ESTs matched to each Gene ID and provides their sequence.** (XLSX 4469 kb) Additional file 2:
**Lists 1) all differentially expressed genes by gene ID with Blast2Go and KOG annotation for each gene; 2) normalized average expression levels for each gene tested in each sample type; 3) adjusted** ***p*** **-values** **for each comparison performed with differentially expressed genes highlighted in red.** Where a significant change was observed, the “expression change” column provides a description of the direction of the change (up or down) and in which tissue type(s) (blastema or opposite side) the change was detected. (XLSX 1831 kb) Additional file 3:
**Provides identifiers for 5′ and 3′ EST sequence reads or primer sequences for clones used to make riboprobes.** “Blastema”, “Neoblasts” and “CNS” columns indicate whether or not each gene was expressed in each of those tissues based on WISH. (XLSX 54 kb) Additional file 4:
**Lists all genes for which RNAi experiments were performed, with identifiers for the 5′- and 3′-end sequence reads or primer sequences for each clone used.** The genes for which knockdown produced a phenotype are marked with the names they are referred to by in Table 1. (XLSX 41 kb) Additional file 5:
**Shows in situ hybridization to the genes (excluding** ***ima-1***) **for which knockdown resulted in a phenotype.** Animals were imaged from the ventral side and are shown with anterior to the left. Inset images for *ddc* and *tph* show photoreceptor expression imaged dorsally with anterior toward the top. (JPEG 583 kb) Additional file 6:
**Shows univariate scatter plots of measurements/counts taken on regenerating** ***Smed-ima-1(RNAi)*** **animals.** Open circles represent values for individual animals and horizontal lines indicate the mean for each group. 6A shows differences in the size of the cephalic ganglia on day ten of regeneration in *Smed-ima-1(RNAi)* compared to *gfp(RNAi)* controls, and 6B shows differences in the number of sensory neurons (*cintillo* ^*+*^ cells) on the same day. (JPEG 214 kb)

## Abbreviations

ARM: Armadillo repeat
cg: Cephalic ganglia
CNS: Central nervous system
dsRNA: Double-stranded RNA
IBB: Importin-β binding domain
NLS: Nuclear localization signal
PH3: Phospho-Histone H3S10
RNAi: RNA interference
SmedGD: *Schmidtea mediterranea* genome database
vnc: Ventral nerve cords
WISH: Whole-mount in situ hybridization
