# Supplementary figures and images for: A functional genomics screen identifies an Importin-α homolog as a regulator of stem cell function and tissue patterning during planarian regeneration
Source: BMC Genomics. 2015 Oct 12;16:769. doi: 10.1186/s12864-015-1979-1 (PMC4603911; doi:10.1186/s12864-015-1979-1)

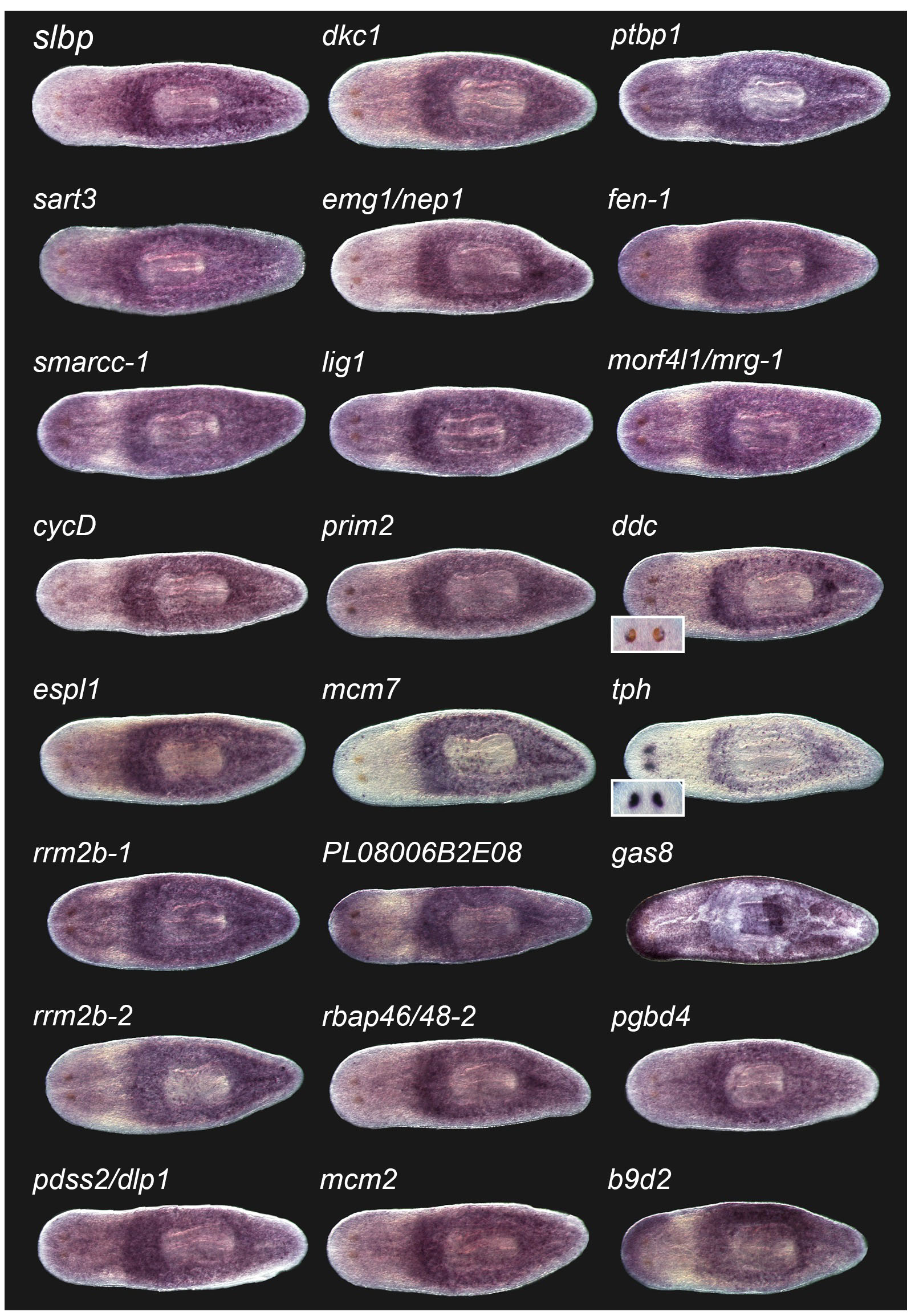

Supplement: Additional file 5: — Shows in situ hybridization to the genes (excluding ima-1) for which knockdown resulted in a phenotype. Animals were imaged from the ventral side and are shown with anterior to the left. Inset images for ddc and tph show photoreceptor expression imaged dorsally with anterior toward the top. (JPEG 583 kb) [file 12864_2015_1979_MOESM5_ESM.jpg]

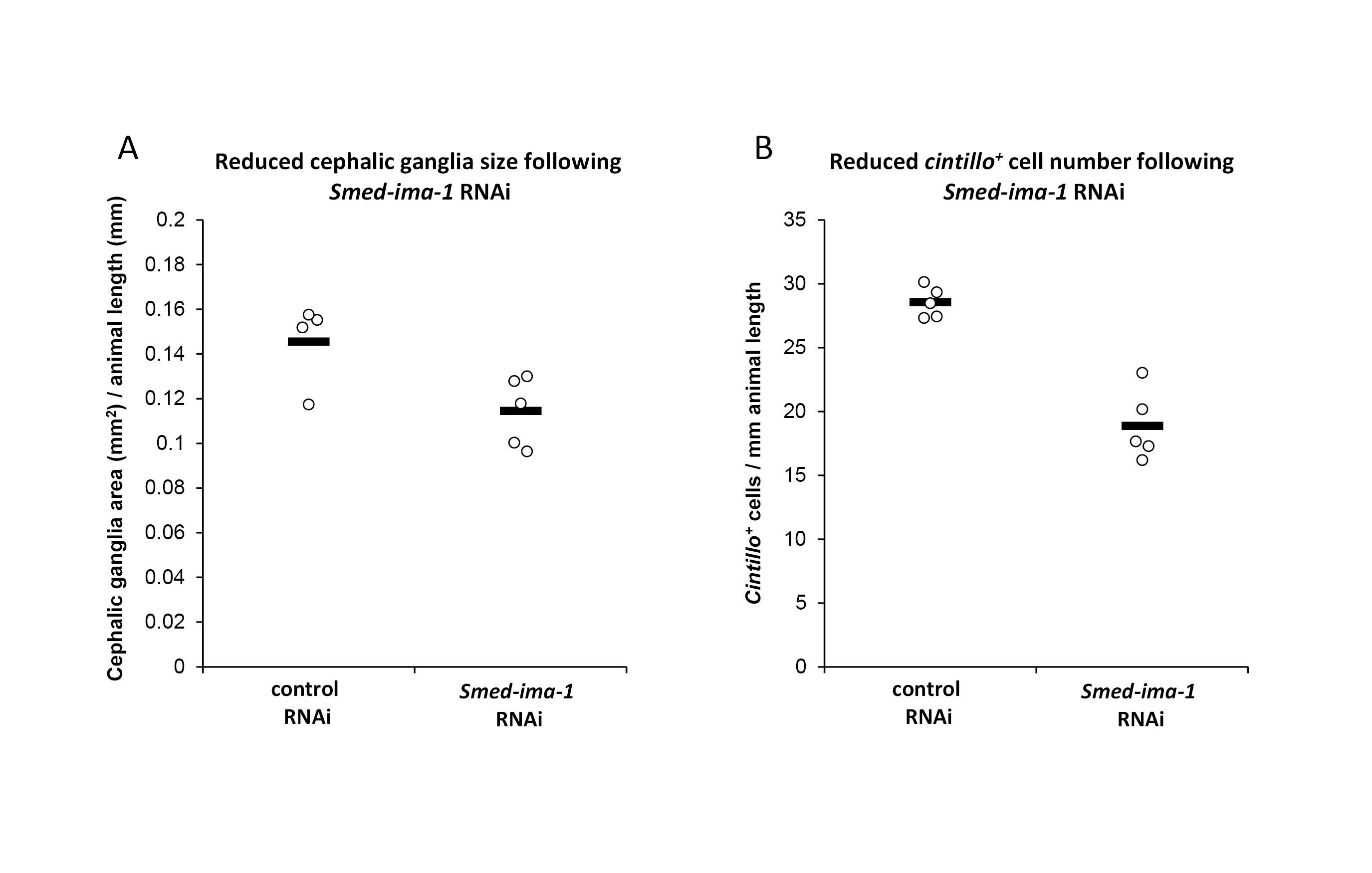

Supplement: Additional file 6: — Shows univariate scatter plots of measurements/counts taken on regenerating Smed-ima-1(RNAi) animals. Open circles represent values for individual animals and horizontal lines indicate the mean for each group. 6A shows differences in the size of the cephalic ganglia on day ten of regeneration in Smed-ima-1(RNAi) compared to gfp(RNAi) controls, and 6B shows differences in the number of sensory neurons (cintillo + cells) on the same day. (JPEG 214 kb) [file 12864_2015_1979_MOESM6_ESM.jpg]
